# Designing a kidney exchange program in Germany: simulations and recommendations

**DOI:** 10.1007/s10100-024-00933-0

**Published:** 2024-08-29

**Authors:** Itai Ashlagi, Ágnes Cseh, David Manlove, Axel Ockenfels, William Pettersson

**Affiliations:** 1 Department of Management Science and Engineering, Stanford University, 475 Via Ortega, Stanford, CA 94305 USA; 2 Department of Mathematics, University of Bayreuth, Universitätsstraße 30, 95447 Bayreuth, Germany; 3 School of Computing Science, University of Glasgow, University Avenue, Glasgow, G12 8QQ UK; 4 Department of Economics, University of Cologne, Universitätsstraße 22a, 50923 Cologne, Germany; 5 Max Planck Institute for Research on Collective Goods, Kurt-Schumacher-Str. 10, 53113 Bonn, Germany

**Keywords:** Kidney exchange, Market design, Simulation

## Abstract

We examine some of the opportunities and challenges concerned with establishing a centralized national kidney exchange program in Germany. Despite the many advantages of a national program, without deliberate design and policy intervention, a fragmented kidney exchange program may emerge. We study a number of collaboration strategies, and resulting simulations suggest that transplant centers may find it advantageous not to fully participate, resulting in a net reduction in the number of transplants. These results also suggest that allowing more forms of kidney exchange, such as three-way exchanges and non-directed donations, can significantly increase the number of transplants while making participation in a national program more attractive and thus national coordination and cooperation more robust. We propose a multi-level policy approach that is easy to implement and would promote an efficient German kidney exchange program that benefits recipients, donors and hospitals.

## Introduction

Kidney transplantation is the optimal treatment for people with end-stage renal disease. However, the demand for kidneys in Germany and globally significantly exceeds the available supply. The alternative to transplantation is dialysis and this is both costly for health systems and significantly reduces the quality of life for patients and their families.

Transplants can use organs from both deceased and living donors. Donations via deceased donors are arranged by Eurotransplant in Germany, but Eurotransplant are currently unable to facilitate living donor transplants. However, living donor transplants are preferred as they lead to better outcomes for recipients (Hart et al. 2017). These living donor transplants are viable because individuals can lead healthy lives with only one kidney. However, not every willing and medically suitable donor can donate directly to their chosen recipient because of the need for blood and tissue type compatibility between donor and recipient. This limitation introduces the concept of kidney exchange, a system in which two or more incompatible donor-recipient pairs exchange kidneys. This ensures that each recipient involved receives a kidney from another donor with whom he or she is compatible. This approach can take several forms, including 2-way and 3-way exchanges, and kidney donation chains initiated by a non-directed donation (Rees et al. 2009; Ashlagi et al. 2012; Anderson et al. 2015). All variations can be very valuable in kidney exchange programs (KEPs) by significantly expanding the pool of available kidneys, increasing the chances of successful transplantation and addressing the critical shortage of organs (Roth et al. 2004, 2005; Roth 2010).

KEPs are prevalent in many countries around the world. For example, there are several KEPs in the USA, including those run by the Alliance for Paired Kidney Donation, the National Kidney Registry and the United Network for Organ Sharing. The Netherlands was the first country to start a nationwide KEP in 2004. Within Europe, the largest KEP (by numbers of participants) is the UK Living Kidney Sharing Scheme, which began in 2007. Scandiatransplant run an international KEP involving Denmark, Finland, Iceland, Norway and Sweden, whilst other groups of countries that run cross-border KEPs include Italy, Portugal and Spain, and also Austria, Czechia and Israel. See Biró et al. (2019) for more information about KEPs across Europe.

At present, in Germany, living kidney donation is permitted only for transplantation to relatives, spouses, cohabiting partners, fiancé(e)s, and others who have a special relationship with the donor. This severely limits the scope of kidney exchange (Kübler and Ockenfels 2020). A proposed reform of living organ donation aims to widen this scope. This raises the question of how kidney exchange should be organized.

Based on an initiative to allow kidney exchange in Germany, this commentary briefly discusses in Sect. 2 some of the benefits of kidney exchange and how an initial program would benefit not only from the simplest form of 2-way exchange but also from expanding a program to allow longer cycles of kidney exchange and non-directed donation in Germany. Enabling both longer exchange cycles and allowing non-directed donation can significantly increase the number of transplants, beyond what simple 2-way exchanges can do, with the largest increase being associated with the introduction of 3-way exchanges. We then show in Sect. 3 that a centralized kidney exchange program has strong advantages, most importantly an increased number of transplants, although—in line with the experiences of some other countries—individual hospitals might have an incentive not to (fully) participate.^1^ We conclude in Sect. 4 with concrete recommendations for the design of the German KEP.

We remark that, although there have been various simulation studies previously that examine the effects of policy decisions on KEPs (see Sect. 2.2 for further details), our paper is tailored to the German application, and is intended to provide policy recommendations. Several of our simulations involving collaboration strategies are based on models for hospitals forming coalitions that may be worthy of further independent study.

A preliminary version of this study was considered by the legislators, and our recommendations are reflected in the draft law, in particular by requiring hospitals to submit all donor-recipient pairs and anonymous non-directed donors to a centralized KEP. Perhaps the most important recommendation for further adaptation of the bill is to allow compatible pairs to participate in kidney exchange, as suggested in the literature (Ockenfels et al. 2024).

## Benefits of allowing variations in kidney exchange

### Background

A simple 2-way kidney exchange occurs when two pairs of living kidney donors and recipients, who are incompatible within their own pairs, swap donors so that each recipient can receive a compatible kidney. Essentially, Pair A’s donor gives a kidney to Pair B’s recipient, and Pair B’s donor gives a kidney to Pair A’s recipient. Expanding on this concept, a 3-way kidney exchange involves three pairs of donors and recipients. Similar to the 2-way exchange, each donor in this scenario is incompatible with their intended recipient but compatible with a recipient in another pair. In a 3-way exchange, Donor A donates to Recipient B, Donor B donates to Recipient C, and Donor C donates to Recipient A. This circular exchange allows for three transplants to occur, all facilitated by the compatible matches that were not possible in the original pairings.

Kidney donation chains are an extension of the kidney exchange concept, initiated by a non-directed donor (also known as an altruistic donor). A non-directed donor is someone who offers to donate a kidney without having a specific recipient in mind. This act can initiate a chain of transplants across multiple pairs of donors and recipients. The process begins when the kidney from the non-directed donor is given to a recipient in need who has a willing but incompatible donor. That recipient’s incompatible donor then gives a kidney to another recipient in a different pair, whose donor then donates to yet another recipient, and so on. The chain can potentially extend to involve numerous pairs, significantly increasing the number of people who receive transplants compared to traditional one-to-one exchanges. It typically ends when the final donor donates to a recipient on a deceased donor waiting list (DDWL).

**Fig. 1:**
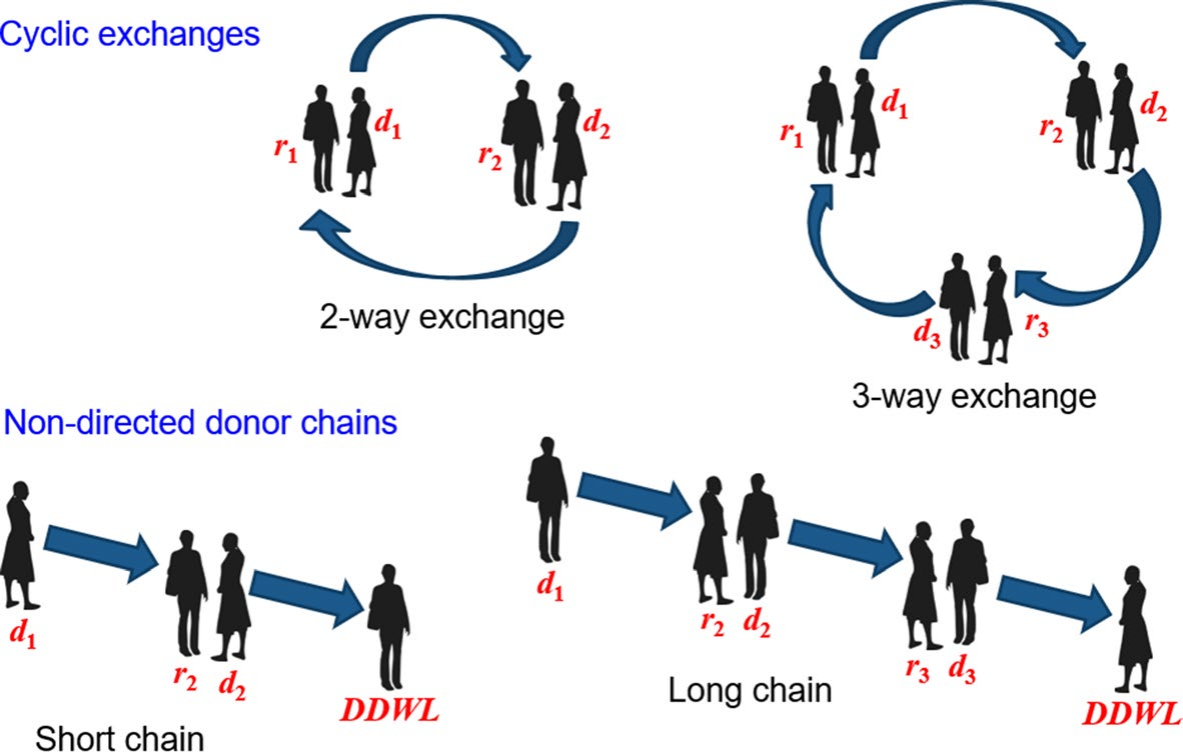
Variations of kidney exchanges. In the case of a 2-way exchange, donor $d_1$ donates a kidney to recipient $r_2$, in exchange for donor $d_2$ donating a kidney to recipient $r_1$. In the case of a 3-way exchange, three donor kidneys are swapped among three recipients in a cyclic fashion. For a short non-directed donor chain, a non-directed donor $d_1$ donates a kidney to recipient $r_2$, in exchange for donor $d_2$ donating a kidney to a DDWL recipient. A long chain is similar but involves one additional donor-recipient pair

Figure 1 provides a graphical illustration of the simple 2-way exchange and its variations.

In this section we study the impact of allowing 3-way exchanges, as well as chains initiated by non-directed donors, on a potential national KEP in Germany. Specifically, we consider four distinct cases:

1. Only 2-way exchanges allowed
2. Only 2-way and 3-way exchanges allowed
3. 2-way and 3-way exchanges as well as non-directed donations involving short chains allowed (a short chain involves a non-directed donor donating to a recipient, and the paired donor of the recipient donating to a DDWL)
4. 2-way and 3-way exchanges, as well as non-directed donations involving short and long chains allowed (a long chain involves a non-directed donor donating to a recipient $r_1$, and the paired donor of $r_1$ donating to a second recipient $r_2$, and the paired donor of $r_2$ donating to a DDWL)

### Simulations

Simulations have oft been used to investigate the impact of changes to KEP policy. Manlove and O’Malley study the effect of increasing the bounds on cycle lengths (Manlove and O’Malley 2015), showing that this can moderately increase the number of identified transplants. Nicolau Santos and Pedroso (2017) give a framework for simulating KEPs that can take into consideration real-world factors such as the risk of a laboratory-based positive crossmatch or recipient dropout. More recent articles have focused on international KEPs (Mincu et al. 2020; Druzsin et al. 2024); in such KEPs there may be different countries with different priorities in terms of criteria and optimality objectives.

To obtain simulated data for Germany, we partly extrapolate from the situation in the United Kingdom (UK). In the UK, there are approximately 5,000 recipients waiting for a kidney transplant, and the UK Living Kidney Sharing Scheme (UKLKSS), the national KEP in the UK, has approximately 250 incompatible donor-recipient pairs in a matching run. This gives a ratio of roughly 20 recipients on a waiting list for each donor-recipient pair in a KEP. Using this and the fact that Germany had 6,689 recipients waiting for a kidney transplant at the end of 2022, we estimate that Germany could have approximately 330 donor-recipient pairs in a KEP. When we examine results for individual transplant centers within Germany, we use information about each hospital’s waiting list and divide the length of the list by 20 to obtain an expected number of donor-recipient pairs within that center.^2^

We also study non-directed donations again extrapolating from the UK experience, where there is approximately one non-directed donor for every 20 recipients. Thus, we estimate that a German KEP could have approximately 15 non-directed donors.^3^

The synthetic data for our simulations were generated using state-of-the-art generators that are themselves based on real historical data from the UKLKSS (Delorme et al. 2022). These generators create instances where a recipient who does not have a blood-group compatible donor has two-thirds chance of being highly sensitized (i.e., having a cPRA $\ge 85\%$), and recipients who do have a blood-group compatible donor have a one-in-four chance of being highly sensitized. In all of our simulations, we study KEPs whose only objective is to maximize the number of identified transplants. We run each simulation 25 times and report the average number of transplants identified across all runs.

Before presenting our results, we caution that the available data on potential donors and recipients in Germany is poor to non-existent, which is why we decided to extrapolate from UK data. Our results should therefore only be taken as a rough indication, without a guarantee of their accuracy. We will return to the lack of relevant data in Germany in our concluding section.

Tables 1 and 2 give an overview of the simulation results; Table 1 shows the total expected number of transplants under a range of scenarios, while Table 2 shows the number of expected transplants to highly sensitized recipients under each scenario. The simulations suggest that if only pairwise exchanges are allowed in a German national program (Scenario A), an average of about 95 transplants will be identified. Allowing 3-way exchanges increases this to 185 on average, an improvement of almost 95%. Further expanding the program by allowing non-directed donations using short chains increases the expected number of transplants to 209, and including long chains improves this to 224. Clearly, the potential benefits of expanding the simple 2-way kidney exchange to allow variations in terms of the number of additional transplants are very large.^4^

One reason is that, when a national KEP allows for more complex forms of exchanges, the program increases the number of potential matches. This occurs because these more complex exchanges can accommodate a greater variety of donor-recipient blood type and antibody mismatches. As a result, the matching algorithm has a larger pool of candidates to work with, increasing the likelihood of finding compatible matches.

An example is that if a donor of pair X can match a specific sensitized recipient of another pair Y, then for a 2-way exchange it is required that the donor of pair Y can also match the recipient of pair X. If this does not happen, the sensitized recipient may not match at all. For 3-way exchanges, the two pairs can still be in an exchange as long as some pair Z can be found such that the donor of pair Y can match its recipient, and the donor of pair Y can match the recipient of pair X.

**Table 1:** Summary of simulation results

	National program only (Scenario A)	Internal first (Scenario B)	Coalitions (Scenario C)
2-way	95	86	89
3-way	185	165	148
3-way + short chain	209	180	166
3-way + long chain	224	181	175

The results are shown in terms of estimated number of transplants across a number of different possible KEP scenarios in Germany. These scenarios are described in detail in the simulations section below

**Table 2:** Summary of simulation results for highly sensitized recipients

	National program only (Scenario A)	Internal first (Scenario B)	Coalitions (Scenario C)
2-way	45	38	39
3-way	111	96	84
3-way + short chain	114	92	82
3-way + long chain	126	96	90

The results are shown in terms of estimated number of transplants to highly sensitized recipients (having a cPRA of at least 85%) across a number of different possible KEP scenarios in Germany. These scenarios are described in detail in the simulations section below

## Benefits and challenges of centralized matching

### Background

There are many advantages to centralized matching in KEPs. Centralized programs can use sophisticated algorithms to optimize matches across a wide range of donor-recipient pairs. This can significantly increase the number of matches and successful transplants, as well as the opportunity for highly sensitized recipients to be matched, compared to decentralized, fragmented matching within individual hospitals. In a centralized setting, organs are allocated to those most in need and most compatible (as determined by the KEP), thus improving overall transplant success rates (Ashlagi and Roth 2014; Toulis and Parkes 2015). A centralized KEP can also be designed to ensure equitable access to transplantation regardless of a recipient’s location, socioeconomic status, or hospital affiliation. Similarly, centralization allows for the implementation of uniform standards and protocols that enhance the safety, ethical integrity, anonymity, and quality of the exchange process.^5^ Finally, another reason for a national KEP to organize the exchange is that if recipients who may participate in the exchange are also on the waiting list for a deceased donor kidney, the data is already in the system.

However, an efficient centralized KEP is often unlikely to emerge “by itself”. Establishing an efficient KEP requires substantial initial investment and maintenance (Cseh et al. 2024), but it must serve all hospitals, donors, and recipients, making free-riding and coordination failures possible or even likely. Even if policymakers or medical associations overcome this collective goods problem and provide the infrastructure for an optimal centralized KEP, not all hospitals may be willing to voluntarily participate fully and submit lists of all their incompatible donor-recipient pairs to the centralized KEP. One reason for this is that it may be more profitable for them to arrange (some) exchanges exclusively within their own recipient base, as this would increase the number of transplants in the hospital—and at the same time reduce the number of national matches that would have been possible with centralized matching.^6^ That is, hospitals may choose to participate in the centralized exchange primarily with their most difficult-to-match pairs, while retaining easier matches for internal resolution. Other reasons for non-participation, which may be important but won’t be discussed further in this commentary, may stem from a lack of uniformity in compatibility assessments, as well as the bureaucratic, financial, accounting, and logistical hurdles associated with enrolling pairs in KEPs.

Selective participation has been identified as a challenge in other countries (Roth 2008). More specifically, incentives to withhold donor-recipient pairs can lead to very costly outcomes in terms of “lost transplants” in small KEPs, but that this need not be the case in large KEPs (Ashlagi and Roth 2014). The theoretical results are mostly based on the assumption that there are no “particularly” large hospitals, which is not the case in Germany. This is why we performed simulations, to provide a first, cautious indication about the scope of the challenges and benefits of potential scenarios that may arise with the introduction of a national KEP within Germany.

### Simulations

We consider four possible ways in which transplant centers could participate in KEPs. In the first, which we call Scenario A, all transplant centers send information on all their recipients and paired donors to a centralized exchange where a centralized matching process is performed. In Scenario B, each transplant center first performs its own internal matching process, and only information on donors and recipients that are not matched in these internal processes is sent to a centralized exchange. Scenario C considers the case where some transplant centers collaborate to form smaller coalitions—we consider a specific such example where Berlin, Hannover, Heidelberg, Essen, and Hamburg each perform their own internal matching process and send unmatched pairs to a central program, while all other transplant centers form a coalition, pool their donors and recipients in an initial matching run, and send only unmatched pairs to a central program. We chose Berlin, Hannover, Heidelberg, Essen, and Hamburg because they are the five largest hospitals by waiting list size.

Our simulations suggest, as shown in Table 1, that the largest number of transplants among our Scenarios A–C can only be achieved when there is a centralized program to which all transplant centers send all of their pairs. This reflects the mathematical necessity that a centralized KEP cannot reduce the number of transplants and will typically increase them substantially. We also see in Table 2 that this same scenario also maximizes the number of transplants to highly sensitized recipients. However, as we show below, there may be situations in which some hospitals are better off without a centralized KEP. This suggests that not all hospitals will necessarily support the development of a national program.

In a further scenario, Scenario D, we examine the effect of a potential coalition of several hospitals, which could include anywhere from 30 to 70% of the pairs within Germany. In this scenario, all centers that are part of the coalition perform a combined matching process and only then send any unmatched pairs to a centralized program. In contrast, all centers that are not in the coalition simply send all of their pairs to the centralized program. Complete results of these simulations are shown in Tables 3, 4, 5, 6, 7, 8, 9, and 10 in our “Appendix”.

When only 2-way exchanges are allowed, our simulations show that such coalitions can sometimes increase the number of transplants within the coalition. Thus, there are cases where individual hospitals or coalitions of hospitals are better off not participating fully in the national exchange. Of course, as before, this strategy comes at the expense of the total number of transplants in Germany.

We note that this type of defection by a transplant center or by a coalition of transplant centers can reinforce incentives for further fragmentation, as defection reduces the value of the exchange program, which may ultimately lead to fewer transplants not only nationally, but even within a single transplant center. To further explore this possibility, we run simulations with the five largest transplant centers in Germany running internal programs, and all other hospitals submitting all donor-recipient pairs to a centralized kidney exchange. Table 11 in the “Appendix” shows that when only 2-way exchanges are allowed, the five largest hospitals running independent programs result in an average of 5.5 fewer transplants in these hospitals in our simulations.

One reason that collective and individual incentives are not necessarily aligned is that in a centralized KEP, highly desirable donors from Hospital X may be matched with recipients from other hospitals, which is optimal from the perspective of maximizing total transplants and potentially improving the quality of matches across the board. However, from Hospital X’s perspective, this means that its “best” donors could be matched to recipients outside its recipient population, which may not be in line with Hospital X’s interest in prioritizing its own recipients.

However, when we allow for other forms of donation, beyond simple 2-way exchanges to 3-way exchanges and chains of donations initiated by a non-directed donor, our simulations suggest that by not fully participating coalitions of hospitals and individual hospitals can only reduce the number of transplants to recipients within the hospital coalition, as well as reducing the total number of transplants (see, e.g., Tables 12, 13, and 14 in the “Appendix”). Collective and individual goals seem aligned in our scenarios.^7^

## Conclusion and recommendations

A centralized national KEP has many important advantages, facilitating transplantation when a willing living donor is incompatible with their intended recipient, thereby significantly expanding the pool of available organs and improving recipient outcomes, reducing waiting times for many recipients on the transplant waiting list (and indeed every recipient matched on the KEP frees up a position on the DDWL), and decreasing healthcare costs.

However, as past experience, incentive theory, and our simulations suggest, a centralized KEP cannot necessarily be expected to emerge by itself. In fact, our simulations suggest that fragmentation in a German KEP can be individually rational for transplant centers, or coalitions of transplant centers, under certain circumstances, while in all scenarios we tested the result of such fragmentation is a net reduction in the number of living donor kidney transplants that could be performed.

We also conclude that allowing more forms of kidney exchange makes participation in a national program more attractive and thus national coordination and cooperation more robust. The result is a double dividend: More flexibility in kidney exchange matching improves the number and quality of transplants because better matches are possible for a given set of pairs in a national program, but also because it increases the number of pairs in the program. One reason is that because a national KEP increases the number of potential matches, as we illustrated earlier, hospitals are more likely to participate in the national program because it offers better opportunities for their recipients. That is, as the KEP becomes more capable of facilitating a variety of exchange types, it becomes inherently more efficient, thereby reinforcing its success.

Based on these findings, we propose a three-step policy to improve a German KEP’s effectiveness, and to prevent the KEP from becoming fragmented, with many lost transplants at both the national and hospital levels ((Ockenfels et al. 2024) provide additional recommendations regarding the matching algorithm that are not covered in this article).

*Step 1 [Establish a National KEP that enables a variety of exchange types]*: The necessary infrastructure is a public good and should therefore be financed and established by the Federal Ministry of Health (BMG) or other public authorities (Cseh et al. 2024) to avoid free-riding and coordination failures. It should be based on state-of-the-art knowledge and scientific literature on matching algorithms for kidney exchange (Biró et al. 2019, 2021), as well as on lessons learned from extensive practical experience in many countries.

In Germany, the most appropriate body to oversee the national KEP would likely be an organization that operates within the existing framework of health care regulation and organ donation systems. Possible candidates include the Bundesärztekammer (German Medical Association), which is the leading national organization of physicians, or the Deutsche Stiftung Organtransplantation (DSO), which is currently responsible for coordinating organ donation in Germany.

Importantly, the kidney exchange and matching algorithm should allow for 3-way exchanges as well as chains of donations initiated by non-directed donors. This can significantly increase the number of matches while mitigating the free-rider problem among hospitals, thereby reducing the need for further interventions as outlined in Step 3.

In addition to maintaining the KEP database, it is desirable to automate data submission by hospitals to ease the burden in typing HLA and antibody data. This has become widely adopted in many KEP programs worldwide.

*Step 2 [Data and Scientific Advisory Board]:* The simplest way to improve effectiveness and to avoid free-riding seems to be to require all hospitals to report all their donor-recipient pairs to a national registry, treating incompatible living donors as a national resource, similar to how cadaveric organs are managed. This would help increase transparency and prevent hospitals from selectively participating in the exchange; optimize matching for the greatest (weighted) number of transplants across the KEP; improve fairness, similar to the principles that govern the allocation of cadaveric organs; increase the likelihood of finding high-quality matches, potentially leading to better outcomes for recipients; and standardize processes for all hospitals, streamlining the exchange process and reducing the potential for errors or inconsistencies.

However, the legislation that is required for mandatory hospital participation would be more complicated to respect the rights and autonomy of donors and recipients while serving the greater public good. In addition, the success of this approach would depend on the cooperation of independent and partly privately operated hospitals and their willingness to comply with the mandate. Therefore, incentives may be needed for hospitals to comply with the KEP and to compensate for the potential loss of control over their transplant programs.

For these reasons, we do not recommend mandatory participation. Instead, however, we strongly recommend regulations requiring hospitals to report all potential donor-recipient pairs to the central KEP, including relevant medical data on donors and recipients, and on which transplants should be done internally versus through the exchange. (This presumably requires the consent of the pairs.) This data should be shared with research organizations and used to monitor the level of free-riding and potential inefficiency (e.g., in terms of lost transplants) in the KEP. All data should be randomly checked for accuracy to avoid errors and misreporting.

We also recommend that an independent Scientific Advisory Board be established to evaluate the data and the exchange program. The board should be closely affiliated to the body that oversees the national KEP (see above) and submit annual reports to the BMG and this body. If the level of free-riding—or other parts of the KEP—are deemed problematic or even unacceptable, the Scientific Advisory Board can make recommendations for improvements to the BMG and this body. For example, Step 3 below suggests measures to discourage hospitals from free-riding and to align hospital interests with the goals of the exchange program.

Regarding data availability, we note that Germany does not have a functioning transplant registry, and recipient participation is explicitly voluntary. This is one reason why, when we contacted several institutions, including the DSO, the German living kidney donation registry SOLKID-GNR and transplant centers, to request relevant data to refine our simulations, we were told that no national data on living donors and (paired) recipients would be available. Solving this problem is both possible, at little cost to hospitals, and necessary, because the lack of data makes it impossible to evaluate and improve a KEP.

*Step 3 [Align incentives]:* If the evaluations in Step 2 suggest that the kidney exchange suffers from hospital free-riding, the measures that we describe here in Step 3 should be considered for implementation. Even if mandatory hospital participation is not feasible, there are several policy measures that can (partially) align hospital interests with the goals of the exchange program. These include a financial reward for full participation in the exchange or for each pair submitted. Similarly, hospitals that help reduce costs by participating in the exchange could receive a share of the savings and thus benefit from system-wide efficiencies.^8^

Alternatively, as a last resort, penalties could be imposed on hospitals found to be withholding compatible pairs. This could include financial penalties, but also reduced access to the exchange.^9^ For example, priority access to better-matched kidneys from the central pool can be given to hospitals that contribute a higher percentage of their pairs. Another form of penalty would be public reporting of free-riding. For example, participation in the centralized exchange can become a metric in hospital performance evaluations, creating a reputational incentive.

More advanced techniques to incentivize hospitals to participate fully include credit systems (Ashlagi and Roth 2014; Klimentova et al. 2021; Benedek et al. 2024), an accounting system for easy and hard to match pairs for each hospital (Agarwal et al. 2019),^10^ as well as matching mechanisms that select transplants based on donor and recipient blood types to maximize the total number of transplants while ensuring that no hospital is worse off than if it had its own internal program (Toulis and Parkes 2015).

We note that beyond the infrastructure and incentives, for hospitals to successfully benefit from KEP it is desirable to further dedicate a nurse coordinator who will engage and educate potential incompatible pairs and non-directed donors about the possibility of exchange (Bingaman et al. 2012; Melcher et al. 2013).

We conclude our commentary by emphasizing that a centralized national KEP can also be more easily linked to other national KEPs to facilitate cross-border kidney exchange than a fragmented KEP. This is because a national KEP has standardized and centralized protocols, data management, communications, and legal and ethical frameworks, making it easier to ensure compatibility and meet the requirements of international partners—especially if all participating countries use state-of-the-art matching protocols. A national entity can also negotiate more effectively with other countries as a single entity, and may be seen as more credible and reliable.

Unfortunately, some international collaborations (e.g., Italy, Portugal, Spain (Valentın et al. 2019)) do not yet function efficiently and instead optimize first nationally and only then report remaining pairs for international exchange (as we described above for hospitals within a German KEP), while others, such as the Scandiatransplant STEP program, involve a truly shared pool (Duus Weinreich et al. 2023)—probably also because each Scandinavian country would have a very small pool on its own.

Germany should establish a robust, well-functioning national KEP that can be easily and straightforwardly integrated into an international KEP.

## Data Availability

For the purpose of open access, the authors have applied a Creative Commons Attribution (CC BY) licence to any Author Accepted Manuscript version arising from this submission. The data used in these simulations is available under a Creative Commons Attribution (CC BY) licence from https://doi.org/10.5525/gla.researchdata.1661. The kep_solver (Pettersson 2022) software used for these simulations is available under the GNU Affero General Public Licence.

## Appendix

Tables 3, 4, 5, 6, 7, 8, 9, 10, 11, 12, 13, and 14.

**Table 3:** Simulation outputs when simulating a coalition of various sizes forming and performing its own internal matching first, when only 2-way exchanges are allowed

Proportion of all recipients in coalition	30.00%	32.00%	34.00%	36.00%	38.00%	40.00%	42.00%	44.00%	46.00%	48.00%	50.00%
National matching only, total transplants	94.24	93.36	93.2	96.24	92.8	96.32	89.04	94.48	91.68	99.76	94.96
National matching only, transplants in large hospital	27.88	31.72	29.96	35.08	35.8	39.24	39.72	41.88	40.8	47.44	48.4
Sequential matching in coalition, transplants in coalition internally	14.16	17.92	16.88	21.68	21.68	26	24.96	28.72	28.24	33.12	34.08
Sequential matching in coalition, transplants for coalition in national	14.84	15.6	14.76	15.32	15.76	15.4	15.88	15.68	15.2	17.08	16.64
Sequential matching in coalition, total transplants for coalition	29	33.52	31.64	37	37.44	41.4	40.84	44.4	43.44	50.2	50.72
Sequential matching in coalition, total transplants nationally	91.52	90.08	89.76	92.32	88.8	92.72	85.52	90.96	87.6	95.2	91.92
Change in number of transplants for coalition	1.12	1.8	1.68	1.92	1.64	2.16	1.12	2.52	2.64	2.76	2.32
Change in number of transplants nationally	− 2.72	− 3.28	− 3.44	− 3.92	− 4	− 3.6	− 3.52	− 3.52	− 4.08	− 4.56	− 3.04

**Table 4:** Simulation outputs when simulating a coalition of various sizes forming and performing its own internal matching first, when only 2-way exchanges are allowed

Proportion of all recipients in coalition	50.00 %	52.00%	54.00%	56.00%	58.00%	60.00%	62.00%	64.00%	66.00%	68.00%	70.00%
National matching only, total transplants	94.96	92	94.56	92.4	93.12	94.24	90.32	92.56	95.84	92.56	94.8
National matching only, transplants in large hospital	48.40	47.64	50.24	51.48	53.6	56.6	56.16	60.04	63.72	62.4	66.52
Sequential matching in coalition, transplants in coalition internally	34.08	36	36.24	38.32	39.84	45.04	45.28	48.88	51.84	52.32	56.96
Sequential matching in coalition, transplants for coalition in national	16.64	14.16	16.88	15.64	15.32	14.72	13.4	13.68	14.48	13.56	12.56
Sequential matching in coalition, total transplants for coalition	50.72	50.16	53.12	53.96	55.16	59.76	58.68	62.56	66.32	65.88	69.52
Sequential matching in coalition, total transplants nationally	91.92	87.84	91.92	88.64	88.72	90.88	86.24	88.64	91.68	89.92	91.12
Change in number of transplants for coalition	2.32	2.52	2.88	2.48	1.56	3.16	2.52	2.52	2.6	3.48	3
Change in number of transplants nationally	− 3.04	− 4.16	− 2.64	− 3.76	− 4.4	− 3.36	− 4.08	− 3.92	− 4.16	− 2.64	− 3.68

**Table 5:** Simulation outputs when simulating a coalition of various sizes forming and performing its own internal matching first, when 2-way and 3-way exchanges are allowed

Proportion of all recipients in coalition	30.00%	32.00%	34.00%	36.00%	38.00%	40.00%	42.00%	44.00%	46.00%	48.00%	50.00%
National matching only, total transplants	179.32	177.76	175.2	177	177	178.76	180.16	180.44	182.72	178.2	184.4
National matching only, transplants in large hospital	55.52	56.16	58.72	63.96	68.88	70.24	76.2	79.88	84.6	87.4	92.2
Sequential matching in coalition, transplants in coalition internally	28.96	29.68	30.36	36.56	40.2	42.12	47.04	52.96	57.56	60.8	64.2
Sequential matching in coalition, transplants for coalition in national	26.44	26.28	28.28	25.88	27.76	27.96	28.12	25.32	25.32	25.96	25.92
Sequential matching in coalition, total transplants for coalition	55.4	55.96	58.64	62.44	67.96	70.08	75.16	78.28	82.88	86.76	90.12
Sequential matching in coalition, total transplants nationally	165.12	163	160.52	159.52	157.6	160.04	159.92	157.68	158.4	155.36	158.92
Change in number of transplants for coalition	− 0.12	− 0.2	− 0.08	− 1.52	− 0.92	− 0.16	− 1.04	− 1.6	− 1.72	− 0.64	− 2.08
Change in number of transplants nationally	− 14.2	− 14.76	− 14.68	− 17.48	− 19.4	− 18.72	− 20.24	− 22.76	− 24.32	− 22.84	− 25.48

**Table 6:** Simulation outputs when simulating a coalition of various sizes forming and performing its own internal matching first, when 2-way and 3-way exchanges are allowed

Proportion of all recipients in coalition	50.00%	52.00%	54.00%	56.00%	58.00%	60.00%	62.00%	64.00%	66.00%	68.00%	70.00%
National matching only, total transplants	184.4	185.4	175.16	181.04	184.48	177.32	177.48	177.16	185.32	178.2	176.24
National matching only, transplants in large hospital	92.2	97.8	94.28	99.48	106.52	106.72	109.36	114.2	123.12	121.56	122.72
Sequential matching in coalition, transplants in coalition internally	64.2	70.44	69.8	71.56	76.56	82.76	85.52	89.16	98.52	99.4	101.56
Sequential matching in coalition, transplants for coalition in national	25.92	25.2	23.72	25.96	26.32	22.12	21.36	22.2	21.32	19.4	18.72
Sequential matching in coalition, total transplants for coalition	90.12	95.64	93.52	97.52	102.88	104.88	106.88	111.36	119.84	118.8	120.28
Sequential matching in coalition, total transplants nationally	158.92	158.36	152.44	156.04	158.56	151.68	152.12	153.64	158.84	154.28	152.48
Change in number of transplants for coalition	− 2.08	− 2.16	− 0.76	− 1.96	− 3.64	− 1.84	− 2.48	− 2.84	− 3.28	− 2.76	− 2.44
Change in number of transplants nationally	− 25.48	− 27.04	− 22.72	− 25	− 25.92	− 25.64	− 25.36	− 23.52	− 26.48	− 23.92	− 23.76

**Table 7:** Simulation outputs when simulating a coalition of various sizes forming and performing its own internal matching first, when 2-way and 3-way exchanges and short chains are allowed

Proportion of all recipients in coalition	30.00%	32.00%	34.00%	36.00%	38.00%	40.00%	42.00%	44.00%	46.00%	48.00%	50.00%
National matching only, total transplants	206.6	206.12	215.24	211.52	211.88	202.88	209.24	210.12	208.4	208.64	206.76
National matching only, transplants in large hospital	61.4	67.56	71.52	74.4	82.56	85.52	84.68	93.12	96.52	99.64	102.8
Sequential matching in coalition, transplants in coalition internally	32.64	38.12	40.48	44.32	49.6	56.48	60.88	66	68.4	67.84	75.24
Sequential matching in coalition, transplants for coalition in national	27.84	29.24	29.52	28.48	30.8	28.08	24.72	26	26.68	28.68	25.08
Sequential matching in coalition, total transplants for coalition	60.48	67.36	70	72.8	80.4	84.56	85.6	92	95.08	96.52	100.32
Sequential matching in coalition, total transplants nationally	176.68	178.2	181.96	177.92	179.52	171.44	175.76	176.08	175.04	173.04	170.64
Change in number of transplants for coalition	− 0.92	− 0.2	− 1.52	− 1.6	− 2.16	− 0.96	0.92	− 1.12	− 1.44	− 3.12	− 2.48
Change in number of transplants nationally	− 29.92	− 27.92	− 33.28	− 33.6	− 32.36	− 31.44	− 33.48	− 34.04	− 33.36	− 35.6	− 36.12

**Table 8:** Simulation outputs when simulating a coalition of various sizes forming and performing its own internal matching first, when 2-way and 3-way exchanges and short chains are allowed

Proportion of all recipients in coalition	50.00%	52.00%	54.00%	56.00%	58.00%	60.00%	62.00%	64.00%	66.00%	68.00%	70.00%
National matching only, total transplants	206.76	214.08	204.92	213	210.08	207.8	210.48	208.6	206.8	206.28	214.84
National matching only, transplants in large hospital	102.8	113.28	111.4	117.48	121.96	123.32	132.8	135	135.8	140.2	149.4
Sequential matching in coalition, transplants in coalition internally	75.24	88.04	86.36	90.12	96.72	97.12	108.8	110.76	111.12	119.2	127.64
Sequential matching in coalition, transplants for coalition in national	25.08	24	23.24	23.44	21.96	22.36	21.48	20.84	21.12	19.04	17.8
Sequential matching in coalition, total transplants for coalition	100.32	112.04	109.6	113.56	118.68	119.48	130.28	131.6	132.24	138.24	145.44
Sequential matching in coalition, total transplants nationally	170.64	176.96	170.52	175.76	174.6	171.16	175.96	174.84	174.92	174.64	180.96
Change in number of transplants for coalition	− 2.48	− 1.24	− 1.8	− 3.92	− 3.28	− 3.84	− 2.52	− 3.4	− 3.56	− 1.96	− 3.96
Change in number of transplants nationally	− 36.12	− 37.12	− 34.4	− 37.24	− 35.48	− 36.64	− 34.52	− 33.76	− 31.88	− 31.64	− 33.88

**Table 9:** Simulation outputs when simulating a coalition of various sizes forming and performing its own internal matching first, when 2-way and 3-way exchanges and short and long chains are allowed

Proportion of all recipients in coalition	30.00%	32.00%	34.00%	36.00%	38.00%	40.00%	42.00%	44.00%	46.00%	48.00%	50.00%
National matching only, total transplants	223.28	216.52	226.28	223.12	228.52	223.24	223.28	219.72	225.28	215.64	220.48
National matching only, transplants in large hospital	69.24	68.68	76.16	80.12	86.08	90.28	92.2	95.6	104.88	103.64	110
Sequential matching in coalition, transplants in coalition internally	41.72	38.96	44.6	52.2	56.92	61.48	61.52	66.48	78.04	74.64	81.32
Sequential matching in coalition, transplants for coalition in national	26.28	27.48	28.36	24.92	27.56	26.52	27.08	27.84	23.48	27.68	24.68
Sequential matching in coalition, total transplants for coalition	68	66.44	72.96	77.12	84.48	88	88.6	94.32	101.52	102.32	106
Sequential matching in coalition, total transplants nationally	190.36	185.2	191.96	187.2	190.96	184	186.4	185	184.96	179	181.08
Change in number of transplants for coalition	− 1.24	− 2.24	− 3.2	− 3	− 1.6	− 2.28	− 3.6	− 1.28	− 3.36	− 1.32	− 4
Change in number of transplants nationally	− 32.92	− 31.32	− 34.32	− 35.92	− 37.56	− 39.24	− 36.88	− 34.72	− 40.32	− 36.64	− 39.4

**Table 10:** Simulation outputs when simulating a coalition of various sizes forming and performing its own internal matching first, when 2-way and 3-way exchanges and short and long chains are allowed

Proportion of all recipients in coalition	50.00%	52.00%	54.00%	56.00%	58.00%	60.00%	62.00%	64.00%	66.00%	68.00%	70.00%
National matching only, total transplants	220.48	227.12	219.24	225.36	221.08	226.6	227	223.72	221.16	221.64	226.2
National matching only, transplants in large hospital	110	117.56	117.16	124.64	128.36	133.88	139.96	141.6	146.16	149.56	156.96
Sequential matching in coalition, transplants in coalition internally	81.32	87.32	90.76	97.64	99.88	107.24	111.56	116.76	124	125.32	134.24
Sequential matching in coalition, transplants for coalition in national	24.68	26.48	23.96	24.04	23.48	20.76	22.64	19.92	17.08	19.72	18.2
Sequential matching in coalition, total transplants for coalition	106	113.8	114.72	121.68	123.36	128	134.2	136.68	141.08	145.04	152.44
Sequential matching in coalition, total transplants nationally	181.08	184.92	181.52	187	179.56	184.92	187.32	182.4	182.76	185.48	190.24
Change in number of transplants for coalition	− 4	− 3.76	− 2.44	− 2.96	− 5	− 5.88	− 5.76	− 4.92	− 5.08	− 4.52	− 4.52
Change in number of transplants nationally	− 39.4	− 42.2	− 37.72	− 38.36	− 41.52	− 41.68	− 39.68	− 41.32	− 38.4	− 36.16	− 35.96

**Table 11:** Simulation outputs when simulating five centers running independent programs and one coalition comprising all other centers, when only 2-way exchanges are allowed

Name	Number of fit patients on kidney waiting list	Expected size of KEP pool	Transplants in centralized exchange	Transplants in sequential (1st stage)	Transplants in sequential (2nd stage)	Transplants in sequential (both stages)	Change in number of transplants
Berlin	788	39.4	11.16	3.76	6.2	9.96	− 1.2
Hannover	639	31.95	9.08	2.56	5.12	7.68	− 1.4
Heidelberg	492	24.6	7	1.52	4.32	5.84	− 1.16
Essen	286	14.3	3.92	0.32	2.76	3.08	− 0.84
Hamburg	267	13.35	3.56	0.56	2.08	2.64	− 0.92
Remaining	4217	210.85	60.56	48.64	11.92	60.56	0
Total	6689	3334.45	95.28	57.36	32.4	89.76	− 5.52

**Table 12:** Simulation outputs when simulating five centers running independent programs and one coalition comprising all other centers, when 2-way and 3-way exchanges are allowed

Name	Number of fit patients on kidney waiting list	Expected size of KEP pool	Transplants in centralized exchange	Transplants in sequential (1st stage)	Transplants in sequential (2nd stage)	Transplants in sequential (both stages)	Change in number of transplants
Berlin	788	39.4	21.76	5.56	8.24	13.8	− 8
Hannover	639	31.95	17.36	3.48	7.56	11.04	− 6.96
Heidelberg	492	24.6	13.84	2.64	6.16	8.8	− 5.2
Essen	286	14.3	7.92	0.52	3.84	4.28	− 2.88
Hamburg	267	13.35	7.56	0.24	3.84	4.08	− 2.52
Remaining	4217	210.85	116.36	89.8	16.44	106.24	− 8.24
Total	6689	334.45	184.8	102.24	46	145.84	− 33.8

**Table 13:** Simulation outputs when simulating five centers running independent programs and one coalition comprising all other centers, when 2-way and 3-way exchanges and short chains are allowed

Name	Number of fit patients on kidney waiting list	Expected size of KEP pool	Transplants in centralized exchange	Transplants in sequential (1st stage)	Transplants in sequential (2nd stage)	Transplants in sequential (both stages)	Change in number of transplants
Berlin	788	39.4	24.2	8.32	6.16	14.48	− 8.72
Hannover	639	31.95	20.52	6.4	6	12.4	− 8.12
Heidelberg	492	24.6	16.08	6	4.2	10.2	− 5.88
Essen	286	14.3	9.44	1.72	3.56	5.28	− 4.14
Hamburg	267	13.35	8.48	1.96	3.12	5.08	− 3.4
Remaining	4217	210.85	129.88	106.2	12.6	118.8	− 11.08
Total	6689	334.45	208.6	130.6	35.64	166.24	− 42.36

**Table 14:** Simulation outputs when simulating five centers running independent programs and one coalition comprising all other centers, when 2-way and 3-way exchanges and short and long chains are allowed

Name	Number of fit patients on kidney waiting list	Expected size of KEP pool	Transplants in centralized exchange	Transplants in sequential (1st stage)	Transplants in sequential (2nd stage)	Transplants in sequential (both stages)	Change in number of transplants
Berlin	788	39.4	26.32	9.16	6.16	15.32	− 11
Hannover	639	31.95	21	7	6.08	13.08	− 7.92
Heidelberg	492	24.6	17.08	4.8	4.12	8.92	− 8.16
Essen	286	14.3	9.4	2.12	2.84	4.96	− 4.44
Hamburg	267	13.35	8.48	1.8	2.56	4.36	− 4.12
Remaining	4217	210.85	142.2	117.16	11.48	128.64	− 13.56
Total	6689	334.45	224.48	142.04	33.24	175.28	− 49.2

## Footnotes

1 As we will argue below, the fact that patients are free to choose their transplant center does not eliminate the problem, because individual hospitals may not fully participate because they want to maximize the number of transplants for their own patients.

2 We caution that our estimates do not take into account that the proportion of live kidney transplants actually performed can vary widely from transplant center to transplant center, and that the proportions under a new system and new incentive schemes are hard to predict.

3 The data for the UK is taken from Delorme et al. (2022). The data for Germany can be extracted from the detailed reports of each center that are available at the website of the “Deutsche Stiftung Organspende”; for the center in Aachen, for instance, see page 13 of https://dso.de/BerichteTransplantationszentren/Aachen%20Niere%202022.pdf. Of course, it remains to be seen whether the frequency will be comparable in Germany, where the issue of living kidney donation is currently more controversial than in other countries.

4 We caution that we identify matches after the simulator generates the entire pool of incompatible donor-recipient pairs and non-directed donors. In particular we ignore here the dynamic nature in Kidney Exchange Programs, in which pairs arrive and matches are conducted over time.

5 National matching also confers advantages in terms of operational efficiency which improves recipient and staff experience. For example, allocation of operating theatres, surgical teams and support services such as High Dependency/Intensive care beds can be planned further in advance. This can greatly enhance the recipient experience—both those who are donating and those who are receiving and will support ancillary aspects of care and recovery, for example, making arrangements for post operative care.

6 For example, suppose there are four donor-recipient pairs, namely pairs A, B, C, and D. Suppose there are two hospitals, X and Y, with pairs A and B under the care of hospital X, and pairs C and D under the care of hospital Y. Suppose that donor A is compatible with recipient B, donor B is compatible with recipient A, donor A is compatible with recipient C, donor C is compatible with recipient D, and donor D is compatible with recipient A. If both hospitals cooperate and report all their pairs to a centralized KEP that seeks to maximize the number of transplants, then three transplants are possible through a three-way exchange involving donor-recipient pairs A, C, and D. However, under this solution, only one of the recipients at hospital X receives a transplant. If, instead, X decides to withhold both of its pairs from a centralized KEP and match them internally, it can achieve a 2-way exchange involving pairs A and B, and thus both of its recipients receive a transplant. However, once this occurs, hospital Y will not be able to obtain any transplants involving its recipients, and thus the overall global social welfare is harmed by X’s strategy. While the experience in Germany so far has shown that matching within a single center is unattractive or difficult for centers due to a lack of digital resources for matching and a small pool of pairs per center, this might change as kidney exchange becomes easier and more popular.

7 While these simulations show that there is no incentive to form coalitions when more variations of kidney exchanges are allowed, we caution that these results may depend on the specific strategies available to hospitals. In our simulations, we assume each coalition first maximizes the number of internal transplants, rather than cleverly optimally determining which pairs should be matched internally and which should not. We cannot rule out the possibility that other strategies (e.g., a coalition first tries to internally match their own hard-to-match recipients) may make it beneficial for such coalitions to form.

8 To improve incentives in the case of chains of donations induced by a non-directed donor (see below), there could be a constraint that a chain ends with a recipient at the donor’s hospital, if necessary.

9 The National Kidney Registry in the US waives an annual membership fee for hospitals that enroll all their pairs https://portal.kidneyregistry.org/docs/NKR_MC_Terms_Conditions.pdf.

10 See also the National Kidney Registry Center Liquidity Contribution Scoring at https://portal.kidneyregistry.org/docs/CLC_Guidelines.pdf.
